# "Influence of methadone on clopidogrel in addicts on methadone maintenance therapy" Drug interaction between methadone and clopidogrel

**Published:** 2016

**Authors:** Ferigol Fallah, Abolhasan Hamidikenari, Seyed Navid Sajadi, Seyed Rohollah Sajadi, Mohammadreza Shiran

**Affiliations:** 1 Psychiatry and Behavioral Sciences Research Center, Mazandaran University of Medical Sciences, Sari, Iran.; 2 Molecular *and Cell Biology* Research Center, Mazandaran University of Medical Sciences, Sari, Iran.; 3 Immunogenetics Research Center, Faculty of Medicine, Mazandaran University of Medical Sciences, Sari, Iran.

**Keywords:** Clopidogrel, Methadone, Interaction

## Abstract

**Background:**

Clopidogrel is a prodrug that converts in the liver to an active thiol metabolite, which irreversibly inhibits the platelet P2Y_12_ adenosine diphosphate receptor. It seems that methadone as CYP2C19 inhibitor affects ticlopidine activity i*n vivo*. This study aimed to test the ability of methadone in changing ticlopidine pharmacokinetics.

**Methods:**

We conducted a case–control study in 10 subjects. The cases (5 subjects) in our study were addicts who were receiving methadone maintenance treatment (MMT) for preventing opium withdrawal symptoms. The control group were opiate users before starting MMT. In both groups, the patients received clopidogrel (75mg/day) for 5 days. On the 6^th^ day, the subjects returned to the clinic, blood samples were taken up to 12 hours following clopidogrel dosing in case and control groups. Plasma concentration of clopidogrel was measured by GC-MAS. Noncompartmental pharmacokinetic analysis was performed using Microsoft Excel software to estimate PK parameters.

**Results:**

In this study, methadone decreased clopidogrel clearance by 25% and increased the AUC_0-inf_ nearly 1.3 fold during the coadministration of clopidogrel as an antiplatelet drug.

**Conclusion:**

A significant decrease in the clearance of clopidogrel during the coadministration of methadone consistent with a decrease in clopidogrel conversion to its active metabolite and this may decrease its efficacy and may have life-threatening consequences for the patients undergoing clopidogerel maintenance therapy.

The antiplatelet drug clopidogrel is an important therapeutic agent that is used concomittant with aspirin or alone in patients with cardiovascular disease particularly after surgery to prevent recurrent of arterial stenosid (1). Clopidogrel is a prodrug that must be converted into an active thiol-containing metabolite before it can express antiplatelet function (2). Pharmacokinetic studies indicated that clopidogrel is converted into its active metabolite by hepatic CYPs in a two-step oxidation process and CYP2C19 substantially contributes to both oxidative steps that generate the active clopidogrel metabolite. Methadone is a synthetic analgesic that is distinguished by its long duration of action, a property that makes it ideal for the treatment of chronic pain and for opioid withdrawal (3). *Lu et al* (4) have shown that methadone is a mechanism-based inhibitor of CYP19. The aim of the present study was to evaluate the effect of methadone as a mechanism based inhibitor of CYPC19 on the disposition of clopidogrel in MMT patients.

## Methods

A case–control study was conducted in 10 subjects with CYP2C19 extensive metabolizer (EM) genotype. The cases (5 subjects) in our study were those undergoing methadone maintenance thereatment (MMT). The control group was the opiate users before starting MMT. All patients gave written consent before recruitment and the study was approved by the Research Ethics Committee of Mazandaran University of Medical Sciences. In both groups, the patients received clopidogrel (75mg/day) for 5 days. On the 6^th^ day, the subjects returned to the clinic and before taking their daily dose of methadone (case group), 5 ml blood samples were provided at different time points of 0.5, 0.7, 2, 4, 8, and 12 hours following taking clopidogrel. In the control group, a 5 ml blood sample was taken at baseline and at the following time points: 0.5, 0.7, 2, 4, 8, and 12 hours following clopidogrel. Plasma concentration of clopidogrel was treated according to the method of Lagorce *et al*. (5) with some modifications. Noncompartmental pharmacokinetic analysis was performed using Microsoft Excel software to estimate clopidogrel PK parameters. Statistical analysis was performed using SPSS for Windows (Version.12, SPSS Inc., Chicago, USA). In all cases, p<0.05 was taken as statistically significant.

## Results

**Figure 1:**
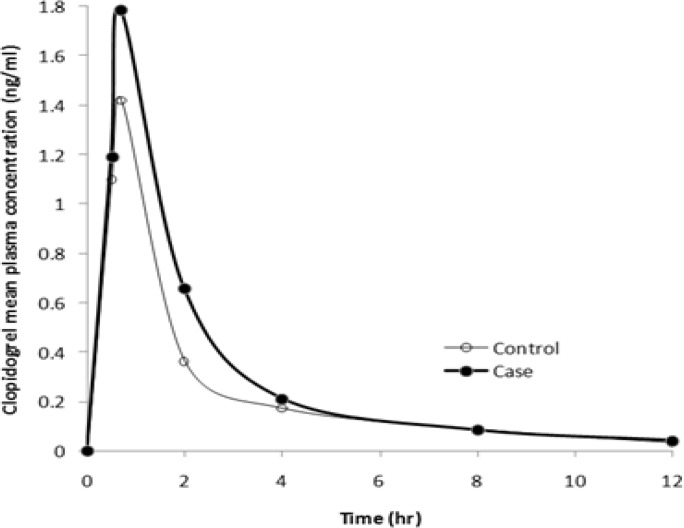
Mean plasma concentration–time profiles of clopidogrel at steady state in case (bold line) and in control treatment groups.

A summary of demographic characteristics of patients along with their drug history is shown in table 1. Pharmacokinetics parameters values for clopidogrel in case and control groups are listed in table ‎2. Both, clopidogrel plasma concentrations (Cmax) and area under the concentration–time curve (AUC0–inf) increased approximately 1.3 fold in MMT patients compared to control group. Although, there was a significant decrease in CL/F values in case group (P=0.03), however, there was no significant increase in t1/2 beta values (P=0.75). The mean concentration-time profile of clopidogrel after administration of clopidogrel 75 mg for five days is shown in fig 1.

**Table ‎1:** Characteristics of subjects who completed the study

**parameters**	**Group**	**Mean**	**SD**
age(years)	Case	34.2	7
	Control	33.5	8.5
Methadone dose (mg/day)	Case	65.4	12
	Control	--	-

**Table ‎2:** Mean pharmacokinetic parameter values for clopidogrel in case and control groups estimated from non- compartmental analysis

	Mean±SD	Ratio of means (case/control)	Pvalue
CL/F (L/hr)			
Case	18760.4±2272.2	0.7	0.03
Control	25227.5±6382.0		
t1/2 beta (hr)			
Case	2.42±0.4	1.0	0.75
Control	2.35±0.3		
AUC 0-inf (ng/hr/L)			
Case	4.0±2.7	1.3	0.54
Control	3.1±2.0		
Cmax (ng/ml)			
Case	1.9±0.3	1.3	0.03
Control	1.4±0.4		

## Discussion

To our knowledge, this is the first study which determined the effect of methadone on the disappearance of pharmacokinetics of the clopidogrel in patients on maintenance MMT therapy. Antiplatelet therapy with clopidogrel is a standard-of-care for the prevention and treatment of atherothrombotic cardiovascular disease and potential for drug–drug interactions, due to CYP2C19 inhibition, is an important clinical challenge in these groups of patients and often requiring careful monitoring. Many studies (12-13) showed that CYP2C19 genetic polymorphism can affect the pharmacokinetic and pharmacodynamic response to clopidogrel. Methadone as an accepted drug for the treatment of chronic pain and for opioid withdrawal in word (3) is a mechanism-based inhibitor of CYP19 (4). In this study, we demonstrated that concurrent administrations of methadone influence on pharmacokinetics parameters of clopidogrel. The observed increases in the AUC of the clopidogrel with concomitant methadone appear to be due to inhibition of CYP2C19 enzyme or differences in p-gp or other CYPs activity between two groups. 

However our sample size in this study was too small and it needs to be confirmed in another case- control study by larger sample size by elucidating clopidogrel active metabolite pharmacokinetics and its pharmacodynamics. 

## Notes

**Funding: **This manuscript is part of Ferigol Fallah’s thesis. This study has been supported by a grant from the Research Council of Mazandaran University of Medical Sciences.
